# Chain Trajectory, Chain Packing, and Molecular Dynamics of Semicrystalline Polymers as Studied by Solid-State NMR

**DOI:** 10.3390/polym10070775

**Published:** 2018-07-15

**Authors:** Shijun Wang, You-Lee Hong, Shichen Yuan, Wei Chen, Wenxuan Zhou, Zhen Li, Kun Wang, Xu Min, Takashi Konishi, Toshikazu Miyoshi

**Affiliations:** 1 Department of Polymer Science, The University of Akron, Akron, OH 44325-3909, USA; sw105@zips.uakron.edu (S.W.); yh35@zips.uakron.edu (Y.-L.H.); sy33@zips.uakron.edu (S.Y.); weichendave@163.com (W.C.); wz35@zips.uakron.edu (W.Z.); zl16@zips.uakron.edu (Z.L.); kw104@zips.uakron.edu (K.W.); konishi.takashi.8c@kyoto-u.ac.jp (T.K.); 2 RIKEN CLST-JEOL Collaboration Center, RIKEN, Yokohama, Kanagawa 230-0045, Japan; 3 State Key Lab of Pollution Control and Resource Reuse Study, College of Environmental Science and Engineering, Tongji University, Shanghai 200092, China; 4 School of Physics and Materials Science & Shanghai Key Laboratory of Magnetic Resonance, East China Normal University, Shanghai 200062, China; mxu1@uakron.edu; 5 Graduate School of Human and Environmental Studies, Kyoto University, Kyoto 606-8501, Japan

**Keywords:** chain trajectory, chain packing, molecular dynamics, semicrystalline polymer, polymer crystallization, solid-state NMR

## Abstract

Chain-level structure of semicrystalline polymers in melt- and solution-grown crystals has been debated over the past half century. Recently, ^13^C–^13^C double quantum (DQ) Nuclear Magnetic Resonance (NMR) spectroscopy has been successfully applied to investigate chain-folding (CF) structure and packing structure of ^13^C enriched polymers after solution and melt crystallization. We review recent NMR studies for (i) packing structure, (ii) chain trajectory, (iii) conformation of the folded chains, (iv) nucleation mechanisms, (v) deformation mechanism, and (vi) molecular dynamics of semicrystalline polymers.

## 1. Introduction 

Crystallization of long polymer chains leads to drastic structural change from random coils in the melt and solution states into folded chains in thin crystalline lamellae [1,2,3,4,5]. Much attention has been paid to understand the chain-folding structure of semicrystalline polymers over the last half century, because chain-level structures include specific information regarding when, where, and how long polymer chains change their own structures during crystallization [5,6,7,8,9,10,11,12,13,14,15,16,17,18,19,20,21,22,23,24,25,26]. Thus, characterization of folded chain structure is a particularly important subject to understand the crystallization process, as well as the mechanism at the molecular level. 

Three or four decades ago, neutron scattering (NS) on ^2^H/^1^H polymers indicated that the radius of gyration (*R*_g_) of polyethylene (PE) crystallized from the melt is close to that in the melt state, and the *R*_g_ in the solution-grown crystals is much smaller than that in the solution state [7]. Furthermore, local folding structures of PE and other polymers have been investigated by NS [8,9,10,11,12], infrared (IR) [13,14,15], morphological observation by transmission electron microscopy (TEM) via surface decoration by oligomers [16], direct visualization of chains [23,24,25], and force detections of single chain by atomic force microscopy (AFM) [26]. However, there are inconsistent views on the chain-folding patterns because of insufficient experimental resolutions. Moreover, most analytical techniques need either mono disperse molecular weight [12], specific morphology [16,23,26], or large supercooling [7,8,9,10,11,13,14,15]. Thereby, it is still difficult to systematically reveal the chain trajectory of semicrystalline polymers crystallized from the melt and solution states in a wide crystallization temperature (*T*_c_) range. The crystallization process of polymers is governed by kinetics. Thereby, lacking in kinetics effects on chain-level structure is critical in understanding polymer crystallization. 

Solid-state (ss) NMR spectroscopy is a sophisticated and powerful means to investigate the structure and dynamics of organic and inorganic systems [27]. Magnetic anisotropic information of chemical shift anisotropy (CSA) and ^2^H quadruple coupling has significantly contributed to understanding molecular dynamics of polymers in both amorphous and crystalline regions. High-resolution NMR is sensitive to intermolecular interactions, packing structure, and conformations of polymers. Dipolar-based ssNMR techniques have successfully revealed the three dimensional structure of peptides, as well as biomacromolecules, in the past decade [28,29,30]. In polymers, molecular motions including time scale and amplitude of re-orientations in the glassy [27], crystalline [31,32,33,34,35,36,37,38,39,40,41,42,43,44,45,46,47], and melt [48,49] states, and local packing [50] and conformation [51] in the glassy state have been elucidated by various NMR techniques. 

Recently, ^13^C–^13^C double quantum (DQ) NMR spectroscopy has been applied to elucidate chain trajectory and packing structures for ^13^C selectively isotopic enriched *isotactic*-Poly(1-butene) (*i*PB1), *isotactic*-poly(propylene) (*i*PP), and poly(L-lactic acid) (PLLA) in the melt- and solution-grown crystals [52,53,54,55,56,57,58,59,60,61,62,63]. ^13^C–^13^C dipolar interaction is governed by internuclear distance, spin topology, and spin number. In the crystalline regions, atomic coordinates of all carbon atoms have been successfully determined by wide angle X-ray diffraction (WAXD) and electron diffraction (ED). Thus, the experimental DQ results were compared with the simulated ones based on ^13^C atomic positions determined by the folding patterns of ^13^C labeled chains in the crystalline regions [52,55,62]. As results, average values of chain-folding number <*n*>, chain-folding fraction <*F*>, and shape and size of folded chains clusters were extracted. Here, we review recent progress for the chain-level structure and molecular dynamics of various semicrystalline polymers by ssNMR. 

## 2. Definition of <*n*> and <*F*> 

**Scheme 1:**
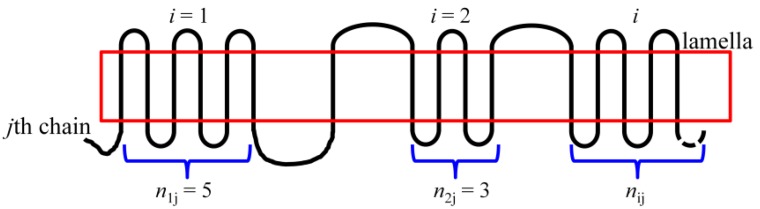
Schematic illustration of chain-folding patterns of *j*th chain in one lamella. *n_ij_* means number of adjacent re-entry folds of the *i*th cluster of the *j*th chain. (Reprinted with permission from *Macromolecules* **2015**, *48*, 5752–5760. Copyright (2015) American Chemical Society).

The chain-folding pattern of the *j*th chain in one lamella is schematically depicted in Scheme 1, where the chain locally forms adjacent re-entry structures. For example, the first and second clusters have five and three adjacent folds, respectively. The number of adjacent re-entry folds of the *i*th cluster of the *j*th chain is defined as *n_ij_*. The term <*n*> can be expressed by an equation of <*n*> = $\sum_{j=1}^{m}(\sum_{i=1}^{lj}n_{ij})/\sum_{j=1}^{m}l_{j}$, where *l_j_* is the number of chain-folding (CF) clusters in the *j*th chain of total *m* chains. *F_j_* is the fraction of the crystallized chain stems participating in the adjacent re-entry clusters in the *j*th chain. Thus, *F_j_* is expressed as $\sum_{i=1}^{lj}(n_{ij}+1)/N_{j}$, where *N_j_* is the total number of crystallized chain stems in the *j*th chain. For example, there are si stems in the CF cluster of *i* = 1 and four stems in cluster *i* = 2. <*F*> is an ensemble average of *F_j_*, which is expressed as <*F*> $=\;\sum_{j=1}^{m}(\sum_{i=1}^{lj}(n_{ij}+1))/\sum_{j=1}^{m}N_{j}$. 

## 3. Packing Analysis

**Figure 1:**
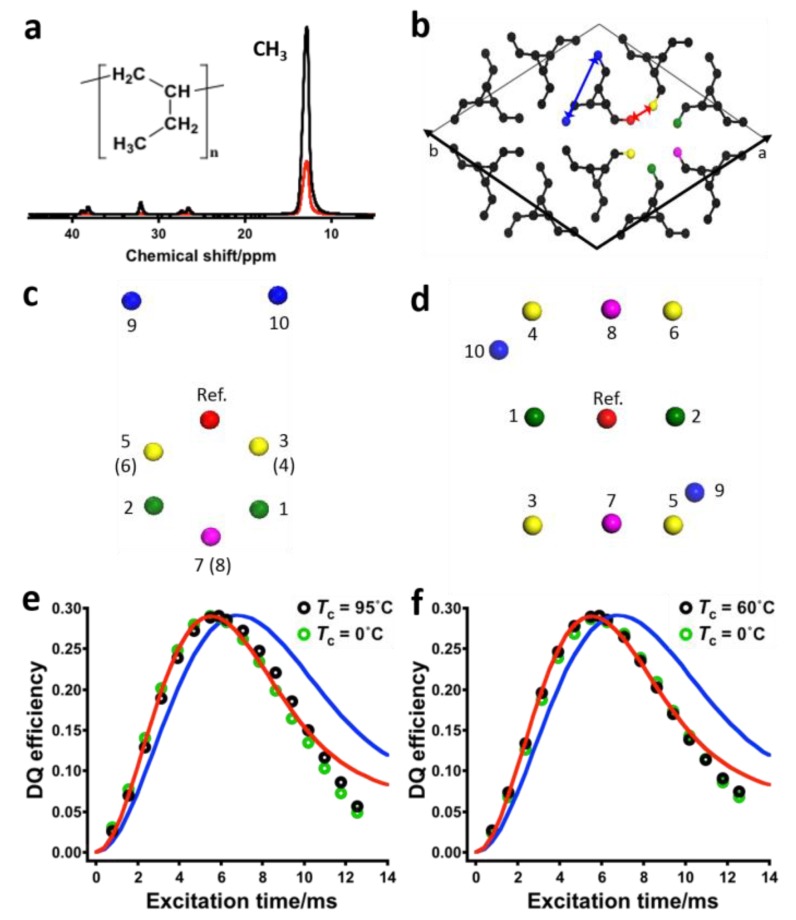
(**a**) ^13^C relaxation-filtered single quantum (SQ) (black) and double quantum (DQ) (red) cross-polarization magic angle spinning (CPMAS) NMR spectra of 35 wt% ^13^CH_3_-enriched *i*PB1 form I-rich sample grown at *T*_c_ = 95 °C. (**b**) Trigonal crystal structure of *i*PB1 form I determined by wide angle X-ray diffraction (WAXD) and corresponding 11-spin system of ^13^C CH_3_ groups on the (001) plane [67]. The projection of the 11-spin systems on the (**c**) (001) and (**d**) (1$\bar{2}$0) planes. The DQ buildup curves of *i*PB1 form I (**e**) melt grown-crystals crystallized at 95 °C (black open circles) and at ~0 °C (green ones) and (**f**) solution-grown crystals at 60 °C (black ones) and at ~0 °C (green ones). In (**e**) and (**f**), the simulated red and blue DQ curves based on the closest interstem ^13^C–^13^C internuclear distance; <*r*> of 4.0 Å; and an exponential *T*_2_ of 8.3 ms and <*r*> = 4.2 Å and *T*_2_ = 10.5 ms, respectively. (Reprinted with permission from *Macromolecules* **2015**, *48*, 3282–3293. Copyright (2015) American Chemical Society).

Figure 1a shows the ^13^C single quantum (SQ) and DQ cross-polarization magic angle spinning (CPMAS) NMR spectra of ^13^C CH_3_ 35 wt% enriched *i*PB1 form I melt-grown crystals at 95 °C [55], acquired using BRUKER AVANCE 300 equipped with a 4 mm double resonance probe at room temperature. The MAS frequency was set to 5102 Hz. The recycle delay and cross-polarization (CP) time was set as 2 s and 1 ms, respectively. A PostC7 sequence pulse [64] with field strength of 35.7 kHz was used for exciting and reconverting ^13^C−^13^C DQ signals into single quantum (SQ) signals. Two-pulse phase modulation (TPPM) [65] and continuous wave decoupling with a field strength of 104 kHz were carried on ^1^H channel during the ^13^C acquisition and recoupling periods, respectively. DQ efficiency (*ξ*) is obtained as the intensity ratio of the DQ and SQ signals. Figure 1e,f depict DQ buildup curves of the ^13^C-labeled *i*PB1 form I melt- and solution-grown crystals, respectively. The initial increase of the DQ curve represents measurement of the apparent dipolar coupling strengths for a given ^13^C site, which represents root mean square (RMS) of many ^13^C–^13^C pair couplings and the latter decay is attributed to *T*_2_ relaxation process. The DQ buildup curve of the melt-grown crystals is identical with that in the solution-grown crystals. Hong et al. quantitatively treated with nuclear magnetic interactions of ^13^C CH_3_ spin systems in the crystalline regions. Eleven-spin system as shown in Figure 1b–d was used to determine the chain-packing and folding structure. The system consists of a reference methyl carbon colored in red as well as the 10 surrounding carbons (eight carbons among interstems and two in an intrastem) at distances of less than 6.4 Å (Figure 1b). The projection of the 11 carbon systems on the (001) and (1$\bar{2}$0) planes is shown in Figure 1c,d. The reference is fixed to detect the DQ signals in the numerical simulations performed using SPINEVOLUTION [55,66]. The atomic coordinates determined by Natta et al. are used for the spin-dynamics simulation [67]. The calculated curve is slightly slower than the experimental curve (Figure 1e,f). Thus, the internuclear distances are slightly shortened by modifying atomic coordinates. The simulated DQ curve based on the shortest ^13^C–^13^C interstem distance of 4.0 Å and relaxation value of *T*_2_ = 8.3 ms can reproduce the experimental data (red curves in Figure 1e,f). Similar packing analysis was conducted on *i*PP [53,54] and PLLA crystals [58,63]. In all systems, the internuclear distances determined by NMR are consistent with those by WAXD and electron diffraction (ED) within 5% errors. 

Very recently, Zhou et al. studied the packing structure of PLLA/poly(D-Lactic-Acid) (PDLA) stereocomplex (SC) by DQ NMR [63]. Ikada et al. reported that PLLA/PDLA blends precipitated by excess poor solvent showed a crystalline structure with a higher melting point (*T*_m_ = 220 °C) than PLLA (or PDLA) homo α crystals (180 °C) [68]. Later on, Lotz et al. proposed the crystal packing structure of SC to be *R*_3c_ or $R_{\bar{3}c}$, where PLLA 3_1_ helices alternatively pack with PDLA 3_1_ helices by paring PLLA and PDLA in a trigonal crystal lattice with *a* = *b* = 1.498 nm, *c* = 0.87 nm, α = *β* = 90°, and *γ* = 120° [69]. Recently, Tashiro et al. carefully investigated fiber WAXD data for SC crystals crystallized from the melt state with different blending ratios of PLLA/PDLA= 7/3–3/7 and proposed the *P*_3_ model [70], where either PDLA or PLLA occupies one site with the probability determined by the blending ratio and the neighboring site can be occupied by either PDLA or PLLA, depending on the blending ratio. However, the calculated WAXD pattern for *P*_3_ model is very similar to that for *R*_3c_. Thereby, Zhou et al. applied DQ NMR to distinguish two structures by atomic resolutions [63]. 

**Figure 2:**
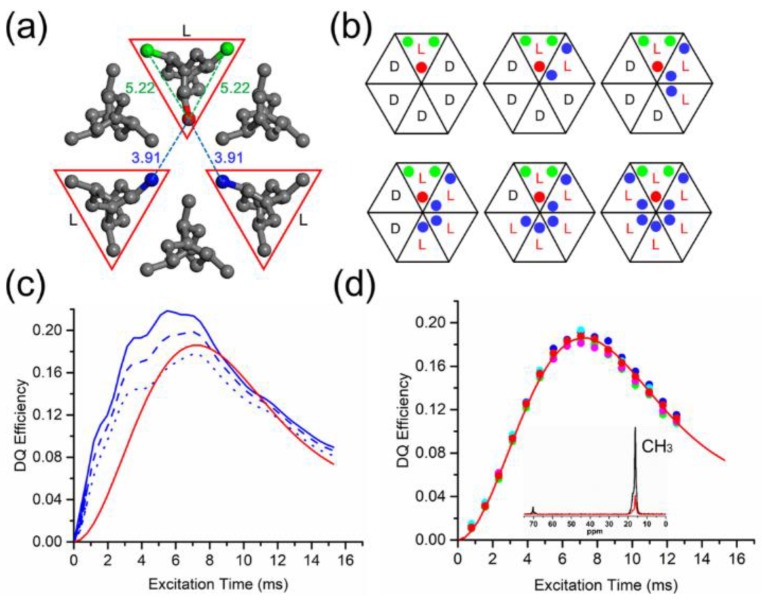
(**a**) *R*_3c_ packing structure and ^13^C five spin systems of ^13^C CH_3_ labeled poly(L-lactic acid) (PLLA) in stereocomplex (SC), including reference (red) two intrastem (green) and two interstem spins (blue) at distance less than 7 Å used in calculations for the DQ curve. (**b**) Schematic drawing for six submodels in the *P*_3_ model. (**c**) Calculated ^13^C–^13^C DQ buildup curves based on *R*_3C_ (red solid) and *P*_3_ models (blue) at blending ratios of PLLA/PDLA=7/3 (solid), 1/1 (dashed), and 3/7(dotted) with *T*_2_ value of 9.5 ms. (**d**) ^13^C CPMAS SQ (black) and DQ (red) NMR spectra for the 33% PLLA/PDLA blends at a blending ratio of PLLA/PDLA= 1/1, with an excitation time of 6.27 ms and DQ buildup curves for PLLA/PDLA SC at PLLA/PDLA blending ratios of 7/3 (pink filled circle), 6/4 (light blue), 5/5 (sky blue), 4/6(light green), and 3/7 (red) with the calculated curve for the *R*_3c_ model. (Reprinted with permission from *ACS Macro Lett*. **2018**, *7*, 667–671. Copyright (2018) American Chemical Society).

In the case of the *R*_3c_ ($R_{\bar{3}c}$) model, the PLLA stems are located at three sites in the hexagons depicted in Figure 2a. A maximum five spin system is statistically treated in simulations of the DQ buildup curve in the *R*_3c_ model [69]. The atomic coordinates determined by ED [69] are slightly different and the shortest inter-stem methyl–methyl carbons distance is shortened to 3.9 Å. The calculated result is depicted as a red curve in Figure 2c. In the *P*_3_ model, PLLA stems are statistically located at each site of the six sites in the hexagon depending on the blending ratio of 7/3–3/7, as shown in Figure 2b. Six kinds of submodels including different PLLA stem numbers were constructed. Thus, the weight-averaged DQ curves for all submodels generate different curves depending on the blending ratio, as depicted in Figure 2b. All three simulated curves for different blending ratios show much faster increases at the initial stage and much larger *ξ*_max_ than the calculated one for the *R*_3c_ model. 

Figure 2d depicts experimental DQ curves of ^13^C-labeled PLLA chains in SC at blending ratios of PLLA/PDLA = 7/3–3/7. The individual curves are very consistent with each other. Mixing-ratio independence of the DQ curves can reject the *P*_3_ model. The results convincingly indicate that PLLA and PDLA stems regularly and alternatively pack with each other. Thereby, DQ NMR with atomic-scale resolution allows one to conclude that PLLA/PDLA SC form *R*_3c_ ($R_{\bar{3}c}$) structure proposed by ED study [69].

Another example is the metastable form of *i*PP. When *i*PP melt is slowly cooled, the stable *α* crystal can be formed. On the other hand, quenching with cooling rates above ~90 K/s results in the mesomorphic form [71]. WAXD data generates two broad maxima at *2θ* = 15.1° and 21.7°. As a result of insufficient resolutions of WAXD pattern, the crystalline structure of *i*PP mesomorphoic form has not been well understood compared with other polymorphs of *α* and *β* forms. To solve this issue, Yuan et al. studied packing structure of *i*PP mesomorphic by DQ NMR approach. By comparisons of the simulated and experimental DQ curves for the *α*, *β*, and mesomorphic form of *i*PP, it is concluded that the packing structure of the mesomorphic form is extremely close to or the same as that for the *β* form [59]. 

## 4. Evaluations of Re-entrance sites of Folded Chains by DQ NMR 

**Figure 3:**
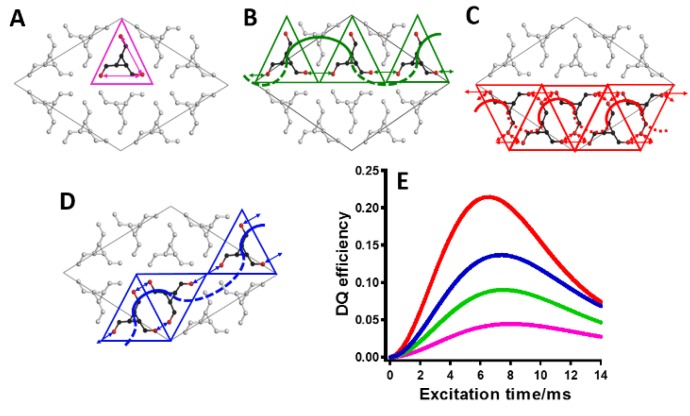
Four chain-folding (CF) patterns of (**A**) CF0, (**B**) CFI, (**C**) CFII, and (**D**) CFIII, and (**E**) the corresponding calculated DQ buildup curves for 35% ^13^C CH_3_ enriched *i*PB1 form I crystal blends with non-labeled *i*PB1 at a blending ratio of 1:9 under the assumption of infinite folding structures [52,55,56]. (Reprinted with permission from *ACS Macro Lett*. **2016**, *5*, 355–358. Copyright (2016) American Chemical Society).

Figure 3A–D illustrates various chain-folding patterns of *i*PB1 in form I crystals. Depending on re-entrance sites of the folded chains, interaction spin number, and spin topology, inter-nuclear distances are varied. Figure 3E depicts calculated ^13^C–^13^C DQ curves for CF0-III of 35% ^13^C CH_3_ enriched *i*PB1 in the form I crystals under the assumption of a blending ratio of labeled and nonlabeled chains with 1:9 [52,55,56]. Infinite chain-folding models are used in the simulations. CFI is an isolated stem without adjacent re-entry structure, where intra-stem ^13^C–^13^C dipolar interactions result in the lowest buildup curve (pink curve in Figure 3E) among the four models. In CFI-III, additional inter-stem ^13^C–^13^C dipolar interactions depending on the re-entrance sites result in faster and higher DQ curves than that in CF0 [52]. For example, the CFII model with a zigzag pattern showed the highest spin density and, as a result, led to the fastest and highest DQ curve (red curve in Figure 3E) among the four models [52]. The simulation perspective emphasizes that the DQ approach potentially identifies different chain trajectories. In real folding analysis, <*n*> and <*F*> in addition to the chain-folding pattern affect experimentally obtained DQ curves.

## 5. Co-Crystallization of ^13^C Selectively Labeled and Non-Labeled Chains at Molecular Scales 

When the isotope labeling technique is applied to structural study in crystalline polymers, special attention must be given to avoid segregations of the labeled and non-labeled chains during crystallization. In fact, slow crystallization of ^2^H and ^1^H PE blends induces physical separations of two components [10]. Thereby, most NS and IR studies using ^2^H/^1^H isotope labeling were conducted on rapidly quenched samples [7,8,9,11,13,14]. This is the reason the kinetics effect on chain-level structure of PE cannot be explored by NS and IR techniques. 

In the literature [55], Hong et al. carefully investigates co-crystallization (segregation) of ^13^C labeled and non-labeled *i*PB1 form I crystals crystallized from dilute solution and melt. The ^13^C–^13^C DQ buildup curves of the *i*PB1 crystal blends with different mixing ratios (10/0, 5/5, 1/9) of ^13^C-labeled/non-labeled *i*PB1 crystallized at 95 °C and solution-grown crystals at 60 °C are measured, respectively. If segregations occur during crystallization, blending-ratio independence of DQ curves is expected. Actually, the mixing-ratio dependences of the DQ buildup curves were clearly observed in both solution- and melt-grown crystals. These results evidently indicate that individual ^13^C-labeled chains are surrounded by the non-labeled chains. The investigated *T*_c_ in both solution- and melt-grown crystallization were the highest. This fact indicates that the ^13^C selective isotopic labeling approach is applicable to chain-folding analysis in both melt- and solution-grown crystals over a wide *T*_c_ range. The same conclusions were obtained in *i*PP [53] and PLLA systems [58]. 

## 6. Solution-Grown Crystals

**Figure 4:**
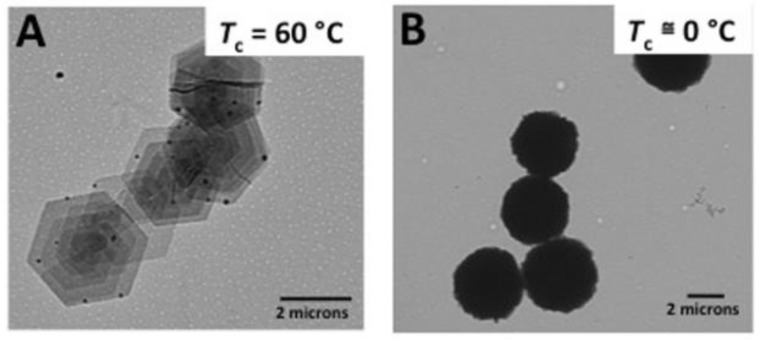
Transmission electron microscopy (TEM) images of *i*PB1(**A**) hexagonal and (**B**) circulated crystals prepared at 60 °C and ~0 °C, respectively, in dilution solution. (Reprinted with permission from *ACS Macro Lett.* **2014**, *3*, 556–559.Copyright (2014) American Chemical Society).

Hong and Miyoshi investigated the morphology and chain-folding structure of *i*PB1 in solution-grown crystals as a function of supercooling [55,56]. Figure 4A,B depicts TEM image of *i*PB1 solution grown crystals, crystallized at 60 and ~0 °C, respectively [55,56]. In the former, the crystals represent hexagonal morphology with a flat face with side-plane length of 2–3 μm and thickness of ca. 6.5 nm (AFM). In the latter, rapid quenching leads to kinetically circulated crystals consisting of multiple layers [56]. The observed morphological change is attributed to kinetics effect. 

**Figure 5:**
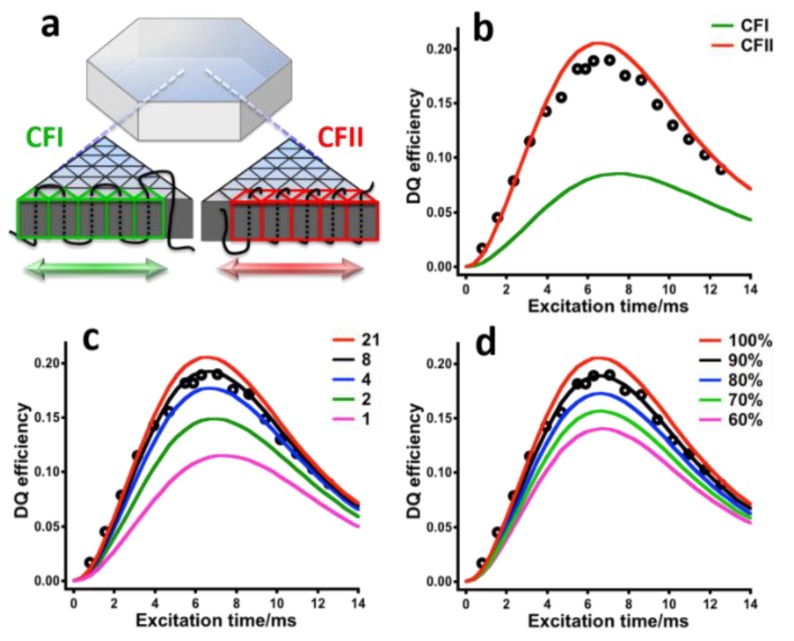
Schematic illustration of (**a**) hexagonal single crystal crystallized in dilute solution at 60 °C and two possible chain-folding patterns of CFI or CFII. (**b**–**d**) Black open circles are the experimental DQ buildup curve for ^13^C-labeled *i*PB1 stems in the blend single crystals with non-labeled chains (1/9) crystallized at 60 °C. (**b**) The simulated DQ buildup curve for CFI and CFII based on <*n*> = 21 and <*F*> = 100%. (**c**) <*n*> and (**d**) <*F*> effects on the simulated DQ buildup curves based on <*F*_CFII_> = 100% and <*n*> = 21, respectively. (Reprinted with permission from *Macromolecules* **2015**, *48*, 3282–3293. Copyright (2015) American Chemical Society).

According to Lauritzen–Hoffman (LH) theory [3,4], it is expected that chain-folding direction is parallel to the growth front at sufficiently high *T*_c_. Thus, CFI and CFII structures are constructed as shown in Figure 5a. On the basis of the crystal thickness of 6.5 nm and the molecular weight, the maximum folding number <*n*_max_> is calculated to be 21. By comparisons of the experimental DQ curve with the simulated curves, it is demonstrated that only the CFII model can reproduce the experimental data at *T*_c_ = 60 °C (black open circle in Figure 5b). After adjusting either <*n*> or <*F*> values, simulated curves based on <*n*> = 21 at <*F*> = 90% and <*n*> = 8 at <*F*> = 100% well reproduce the experimental data (black open circle) as shown in Figure 5c,d [55,56]. 

**Figure 6:**
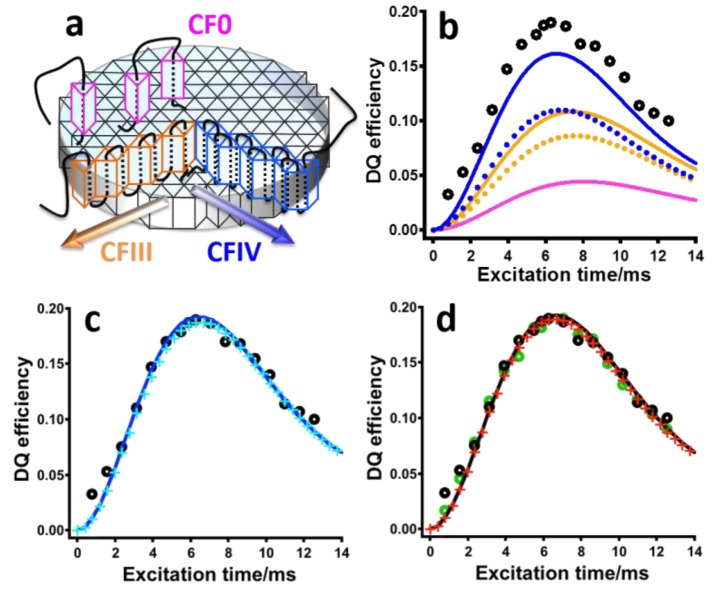
Schematic illustrations of (**a**) the circular crystal crystallized in dilute solution at ~0 °C and three possible chain-folding structures of CF0, CFIII, and CFIV. (**b**–**d**) The black and green open circles represent the experimental DQ buildup curves for ^13^C-labeled *i*PB1 chains in the crystal blends with non-labeled chains (1/9) crystallized at ~0 and 60 °C, respectively. (**b**) The simulated DQ curves for CFIII (orange) and CFIV (blue) with <*n*> = 10 and <*F*> = 80% (solid line) and <*n*> = 5 and <*F*> = 50% (dots) and for CF0 (pink solid curve). (**c**) Blue solid curve is simulated on the basis of CFIV <*n*> = 21 and <*F*_CFIV_> = 95%, and the curve composed of sky blue crosses is simulated on the basis of <*n*> = 8 and <*F*_CFIV_> = 100%. (**d**) Red crosses and black solid curve are the simulated curves for CFII with <*n*> = 21 and <*F*_CFII_> = 90%, and <*n*> = 8 and <*F*_CFII_> = 100%, respectively. (Reprinted with permission from *Macromolecules* **2015**, *48*, 3282–3293. Copyright (2015) American Chemical Society).

Surprisingly, the experimentally obtained DQ curve at *T*_c_ = ~0 °C (open circle in Figure 6b) is well consistent with the DQ curve at 60 °C. This result indicates that at low concentrations (0.03 wt%), the majority of the individual chains adopt nearly perfect adjacent re-entry structures and create clusters of single molecules at ~0 °C, and the cluster size is independence of *T*_c_ used in the experiment [55,56]. This result means that chain-folding event is a local process and is not affected by available kinetics, while kinetics significantly affects morphology. In other words, kinetics differently influences formations of polymer crystals at different length scales. This explanation contradicts the LH theory [3,4], which hypothesizes that microscopic chain-level structure, dominated by kinetics, determines the macroscopic morphology. This is the first experimental data to support nanocluster formation in solution grown crystals. 

It is believed that secondary nucleation generates two dimensional (2D) clusters (linear) of the folded chains under sufficiently low supercooling, while primary nucleation results in 3D clusters to minimize surface free energy [1]. The *T*_c_ independence of cluster size may give one new question about the chain-folding process: *When do polymer chains fold during the crystallization process?* The key issue to answer this question is determination of the cluster shape (2D vs. 3D). Hong and Miyoshi simulated DQ buildup curves for multiple layers nanoclusters for ^13^C-labeled *i*PB1 form I crystal blends with non-labeled chains. Unfortunately, the DQ curve at a single resonance of *i*PB1 form I cannot distinguish between the 2D and 3D clusters [55,56]. 

**Figure 7:**
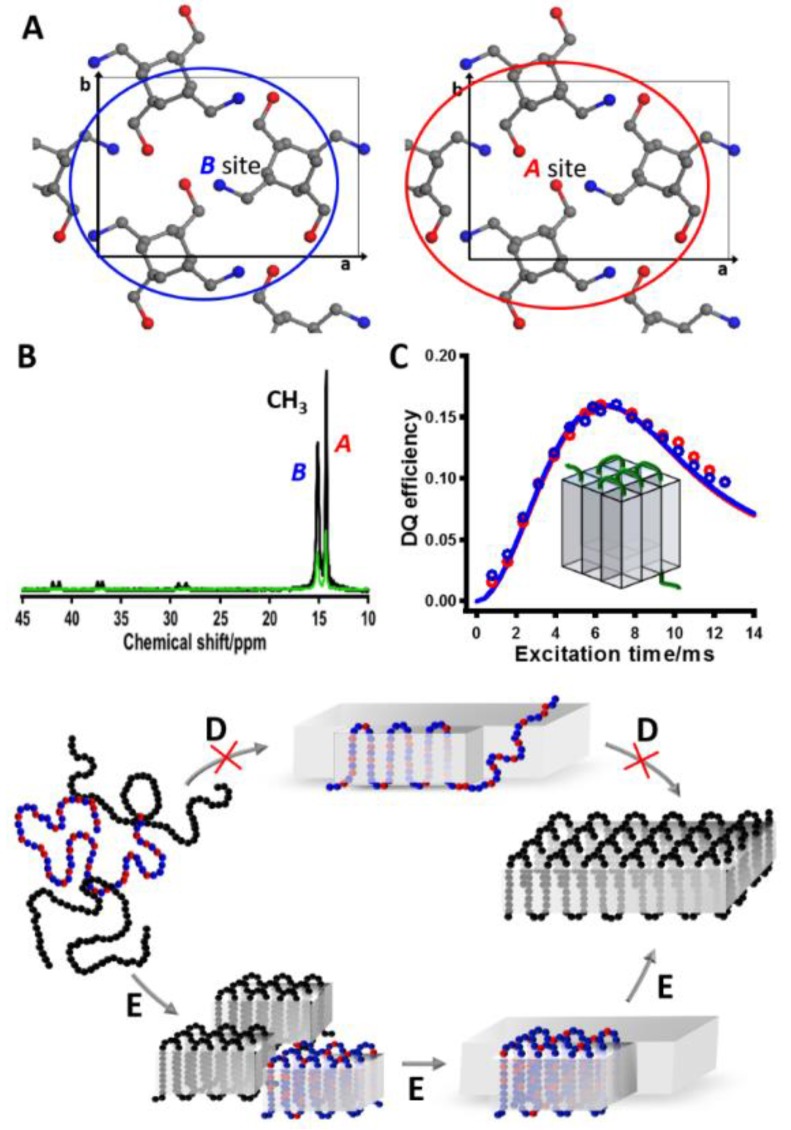
(**A**) Orthorhombic packing structure of *i*PB1 form III and two magnetically inequivalent CH_3_ sites colored by blue (**B**) and red (**A**). (**B**) *T*_1ρH_ filtered ^13^C CPMAS (black) and DQ (green) NMR spectra for 35% ^13^C CH_3_ enriched *i*PB1 form III single crystal blends with nonl-abeled chains at a blending ratio of 1:9, crystallized in dilute solution at 50 °C. (**C**) The DQ buildup curves for the ^13^C labeled CH_3_ signals at A (red) and B (blue). The corresponding calculated curves based on the 3D clusters including twelve stems in four layers at <*F*> = 65% [57]. Two possible chain-folding mechanisms: (**D**) primary nucleation in solution and (**E**) secondary nucleation on the growth front [57]. (Reprinted with permission from *ACS Macro Lett.* **2016**, *5*, 355–358. Copyright (2016) American Chemical Society).

To elucidate the cluster dimensions of the folded chains, as well as the folding mechanism, Hong et al chose form III of *i*PB1 among the various semicrystalline polymers [57]. Form III adopts an orthorhombic packing structure leading to magnetically inequivalent doublet ^13^C peaks for each carbon (Figure 7B). Thus, dipolar interactions at two sites within the same chains are used to recognize either 2D or 3D clusters. By assuming various 2D conformations, simulated data cannot simultaneously reproduce the experimental DQ buildup curves at two sites. On the other hand, 3D clusters in multiple layers, for example, 14 stems in two layers and 12 stems in four layers, can reproduce the experimental data by adjusting the <*F*> value (Figure 7C) [57]. The observed *T*_c_ independence of the 3D cluster structure supports; (i) poor solvent–polymer interactions leads to 3D clusters by self-folding in the initial stage and (ii) the formed clusters are deposited on the growth front as the second step (Figure 7E). The proposed mechanism provides a falsification of the well-known LH theory [3,4], but is consistent with the bundle [6,17,18] and aggregation models [19].

Very recently, Wang et al. investigated morphology, chain-packing structure, and chain-folding patterns of ^13^C 30% CH_3_ labeled PLLA in solution-grown crystals at *T*_c_ = 90, 50, and ~0 °C [58]. At the highest *T*_c_, lozenge shape single crystals with well-defined growth faces are obtained while the lowest *T*_c_ induces dendrite morphology. Again, it is demonstrated that kinetics significantly affects morphology at μm. Interestingly, PLLA chains adopt thermodynamically stable α crystals in solution grown crystals at all *T*_c_ s of 90–0 °C. 

**Figure 8:**
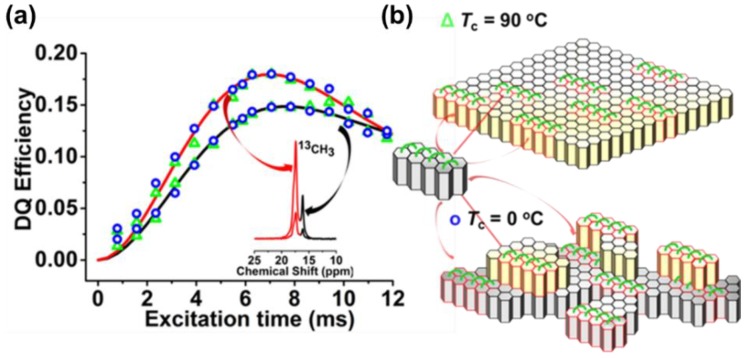
(**a**) ^13^C–^13^C DQ buildup curve for ^13^C CH_3_-enriched PLLA single crystal blends with non-labeled chains crystallized (**a**) in 0.05 wt% amino acetate solution at 90 °C (green open triangles) and ~0 °C (blue open circles). The solid curves with <*n*> = 7 and <*F*> = 100% in two layers represents best-fit curves with experimental data. (**b**) Schematic illustration of nano building blocks of eight stem clusters in solution grown crystals at *T*_c_ = 90 and ~0 °C. (Reprinted with permission from *Macromolecules* **2017**, *50*, 6404–6414.Copyright (2017) American Chemical Society).

By comparisons of experimental DQ curves with simulated ones, only multiple layers model with <*n*> = 7 in two or higher layers can reproduce the experimental data (Figure 8a). Each PLLA chain participates in small nano-clusters in three dimensions. This result also supports three dimensional nanoblock formations of a part of polymer chains prior to crystallization as illustrated in Figure 8b. Nano-block size is much larger than the unit cell of α crystals. *T*_c_ independence of nano-cluster size could reasonably explain why PLLA chains adopt thermodynamically α packing structure even in different morphologies as a function of *T*_c_.

## 7. Melt-Grown Crystals 

Polymer chains are highly entangled in the highly condensed melt. Thereby, topological constraints might significantly affect chain-folding events during crystallization as a function of kinetics. In the literature [52], chain trajectory of *i*PB1 form I crystallized at a wide range of *T*_c_ of 95–~0 °C was investigated by ssNMR. Melting temperature of *i*PB1 is ca. 126 °C. Crystallization at *T*_c_ of 95 °C was really slow and took two days. Small-angle X-ray scattering (SAXS) indicated that crystal lamellae thickness varies between 10 nm at *T*_c_ = 0 °C and 21 nm at *T*_c_ = 95 °C.

**Figure 9:**
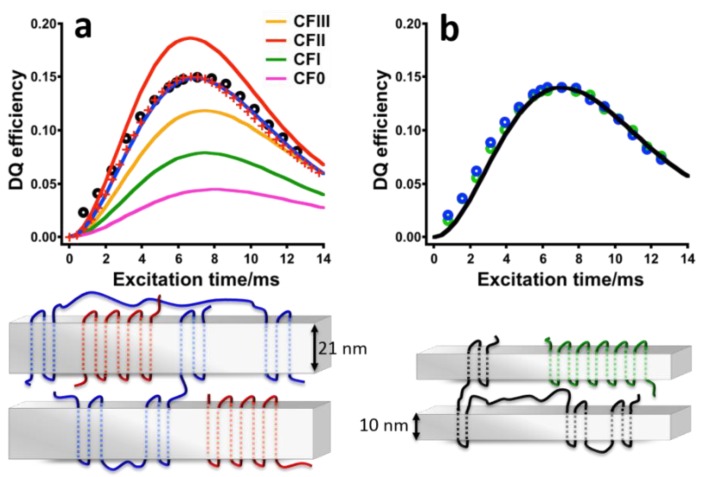
^13^C–^13^C DQ buildup curves for 35% ^13^C–CH_3_ enriched *i*PB1 crystal blends with a non-labeled one with a mixing ratio of 1/9, formed at (**a**) 95 °C (black open circles), (**b**) 50 °C (blue open circles), and ~ 0 °C (green open circles). (**a**) The calculated DQ buildup curves based on CF0 (pink), CFI (green), CFII (red), and CFIII (orange) under the assumption of <*n*> = 5 and <*F*> = 100%. The calculated DQ curves for CFII with <*n*> = 2 and <*F*_CFII_> = 100% (blue solid curve), and <*n*> = 5 and <*F*_CFII_> = 80% (red crosses). (**b**) The calculated DQ buildup curves for CFII with <*n*> = 1.7 with *F*_CFII_> = 100% (black solid) at *T*_c_ = 50 °C and ~0 °C. Schematic illustrations of plausible chain-folding patterns in the lamellae: <*n*> = 5 (red chain) and <*n*> = 2 (blue) on the bottom of (**a**) and <*n*> = 10 (green) and <*n*> = 1.7 (black) on the bottom of (**b**). (Reprinted with permission from *Macromolecules* **2015**, *48*, 3282–3293. Copyright (2015) American Chemical Society).

Figure 9a depicts the DQ buildup curve (black open circles) of 35 wt% ^13^C–CH_3_ enriched *i*PB1 form I crystal blends with non-labeled chains crystallized at 95 °C. The *ξ*_max_ value of 15% is considerably higher than the simulated curves of CF0, CFI, and CFIII, and is lower than that of CFII under the assumptions of <*n*> = 5 and <*F*> = 100%. Thus, only CFII is a plausible structure at *T*_c_ = 95 °C. Using the crystal thickness and molecular weight, the best-fit curves were obtained in two limit structures of <*n*> = 2 and <*F*_CFII_> = 100% (blue solid curve) and <*n*> = 5 and <*F*_CFII_> = 80% (red crosses) (Figure 9a). The possible two chain trajectories in the melt-grown crystals are represented at the bottom of Figure 9a. One structure includes five successive folds in one lamella and another limit structure is a small nano-cluster including three stems connected by two folds. Note that the DQ results cannot distinguish between two limit structures. The *ξ*_max_s of the DQ buildup curves at *T*_c_ = 50 (blue open circles) and ~0 °C (green) are ca. 14% as illustrated in Figure 9b. They are slightly lower than the *ξ*_max_ at *T*_c_ = 95 °C (15%). The best-fit DQ buildup curves to the experimental data at *T*_c_ = 50 and ~0 °C are drawn using CFII with <*n*> = 1.7 and <*F*> = 100% (black solid curve in Figure 9b). This means that the CF structure is almost independent of *T*_c_, even though lamellae thickness drastically changes as a function of *T*_c_.

**Figure 10:**
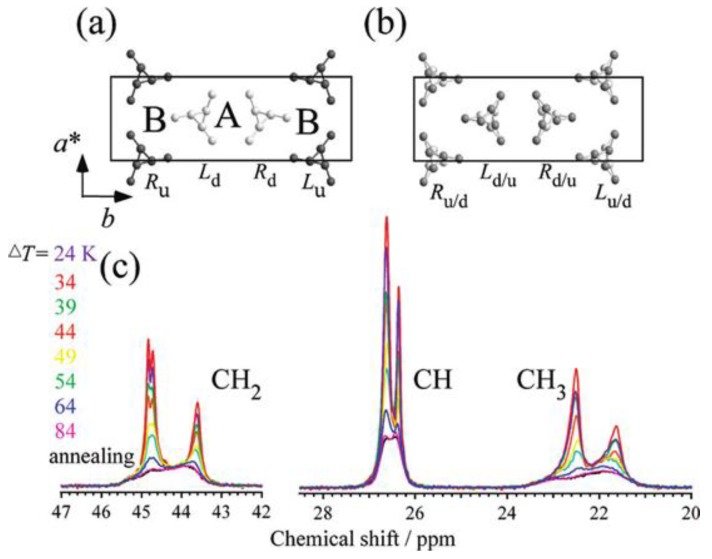
Unit-cell structures of (**a**) α_2_ and (**b**) α_1_ forms of *i*PP. (**c**) The ^13^C CPMAS NMR spectra for the pure α form of *i*PP at different supercoolings. (Reprinted with permission from *J. Phys. Chem. B* **2010**, *114*, 92–100. Copyright (2010) American Chemical Society).

Another interesting sample is *i*PP α crystal. When *i*PP melt is cooled down at temperatures from melt state, *i*PP crystallizes as α form. The α form is further divided into α_1_ and α_2_ crystals as shown in Figure 10a,b [41,72]. The α_1_ crystals are kinetically favored, while the α_2_ crystals are thermodynamically favored. XRD has been used to distinguish α_1_ from α_2_ structure. However, very minor differences led to difficulty of quantification of α_2_ fraction at high *T*_c_.

Figure 10c depicts ^13^C CPMAS NMR spectra for *i*PP melt-grown crystals formed as a function of supercooling [41]. The annealed and low *T*_c_ samples represent structure-less broad ^13^C signals, corresponding to α_1_. The observed broad signals originate from inhomogeneous line broadening due to variations of up- and downward chain orientations. At Δ*T* = 64 °C, narrowed doublet signals appear on the broad line shapes, and the doublet signals grow with decreasing Δ*T*. The observed doublet line shape corresponds to local ordering of the packing structures, namely, α_2_. The fraction of α_2_ can be determined in terms of subtraction of the structure less line shape from the observed spectra at various Δ*T*s. As results, the maximum α_2_ fraction was determined to be 62% at *T*_c_ = 150 °C, where crystallization takes more than 30 days.

Furthermore, the kinetic effect on chain-folding structure was investigated for ^13^C 30 wt% CH_3_ enriched *i*PP (<*M*_w_> = 172 K g/ mol) α_1_ and α_2_-rich crystals, which were crystallized at 100 and 150 °C [41]. It was expected that α_1_ and α_2_ crystals have largely different <*n*> and <*F*>. However, DQ experiments clearly demonstrate that the *i*PP chains adopt <*n*> = ~5 under the assumption of <*F*> = 100% in both α_1_- and α_2_-rich crystals.

Both *i*PP and *i*PB in the melt-grown crystals indicate that <*n*> and <*F*> do not vary as a function of *T*_c_. These newly obtained results contradict expectations from the LH theory [3,4] and indicate that chain-folding events are not related to overall crystal growth rate. It is worthwhile to note that recent MD simulation of coarse grained Poly(vinyl alcohol) (PVA) model predicted <*n*> to be ~2.4 in the melt-grown and <*n*> is independent of *T*_c_ [73,74]. These simulation results are very consistent with recent ssNMR observations [52,53,54,55,56]. 

**Figure 11:**
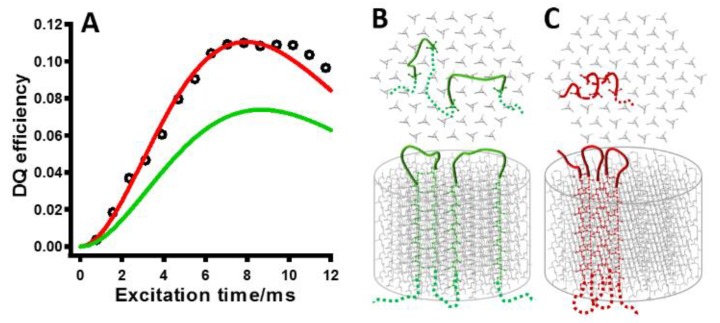
(**A**) ^13^C–^13^C DQ buildup curve for 15 wt% ^13^C CH_3_ enriched *i*PP mesomorphic blends with nonlabeled one with a blending ratio of 1:9. The simulated DQ curves were obtained on the basis of (**B**) isolated model (green) and **(C)** nanocluster model via folding with <*n*> = 4 and <*F* > = 100% [54,62]. (Reprinted with permission from *ACS Macro Lett.* **2016**, *5*, 355–358. Copyright (2016) American Chemical Society).

Understanding the formation of nucleation in the early stages of crystallization is a challenging issue in polymer crystallization. Simultaneous measurements of SAXS, WAXD, and IR enabled us to observe structural evolution of semicrystalline polymer prior to crystallization. Kaji et al. [20] and Olmsted et al. [21] proposed spinodal decomposition (SD) prior to nucleation and growth. It is difficult for DQ NMR to conduct in-situ observations. However, a rapidly quenched system can retain the structure dominated by primary nucleation. One good sample is the *i*PP mesomorphic form obtained by rapid quenching into icy water from the melt. Yuan et al. investigated the chain-folding structure of the mesomorphic form of *i*PP. [54] The mesomorphic domain includes only ~60 stems and its size is comparable to critical nuclei in the initial stage of crystallization predicted by MD simulation. [54] Figure 11A depicts the DQ buildup curve for 15% ^13^C CH_3_ enriched *i*PP mesomorphic blends with non-labeled *i*PP and simulated curves on the basis of isolated model, as depicted in Figure 11B, and nanoclusters (Figure 11C). The comparison indicates that the *i*PP chains fold 3–4 times during rapid quenching into icy water. [54] From the NMR observations, it is concluded that the *i*PP chains initiate nucleation via self-folding in the early stage of crystallization. 

## 8. Molecular Dynamics in Semicrystalline Polymers 

Molecular dynamics of polymer chains in the crystalline regions plays a significant role in polymer crystallization, phase transition, surface melting, mechanical property, and deformations. Among various characterization tools and ssNMR especially, exchange NMR has successfully contributed to understanding detailed dynamics mode and dynamics frequency (or correlation time, <*τ_c_*>) of various semicrystalline polymers, including polyethylene [31,32,33,34,35,75], *i*PP [41,59,72,76], poly(acrylonitrile) [36,77], poly(ethylene oxide) [46], poly(oxymethylene) [78], poly(ethylene terephthalate) [79], *isotactic*-poly(4-methyl-1-penetene) (*i*P4M1P) [38,39,40], PLLA [42,43], Nylon 66 [80,81], Poly(ε-caprolactone) [44,45], poly(tetrafuloroethylene) [82], poly(vinylidene fluoride) [83], and so on. Through NMR dynamics works, one dimensional exchange NMR using center-bands only of detection of exchange (CODEX) NMR [37] has significantly contributed to progress of our understanding molecular dynamics of chemically complex semicrystalline polymers in the last decade [38,39,40,41,42,43]. The CODEX experiments utilize the recoupling of the chemical shift anisotropy (CSA) interaction by 180^o^ pulse trains in the two evolution periods sandwiching a mixing period, *t*_mix_. The dephasing of magnetization brought about by changes in orientation dependent CSA because of a re-orientational dynamic process during *t*_mix_ leads to signal decays in the spectrum (so called exchange spectrum, *S*). Additionally, reference spectrum without dynamics effect (*S*_0_) can be obtained by interchanging *t*_mix_ with a z-filter time after second evolution time in the CODEX exchange pulse program [37].

*i*P4M1P is a unique polymer because crystalline density (0.823 g/cm^3^) is lower than that of the amorphous density (0.840 g/cm^3^) at room temperautre. Dynamics mechanical analysis showed segmental motions in the amorphous regions at around 20–50 °C [38,39,40].

**Figure 12:**
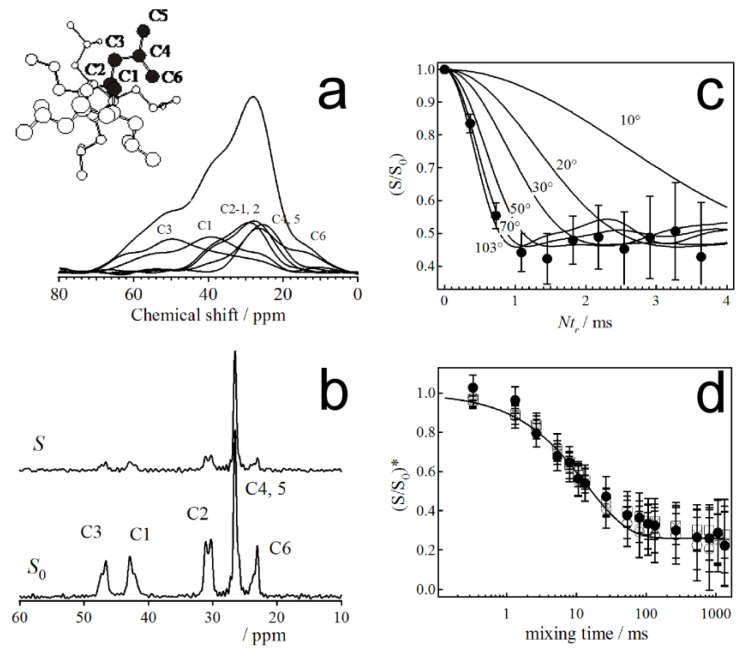
(**a**) *i*P4M1P 7_2_ helical structure and corresponding chemical shift anisotropy (CSA) patterns. (**b**) Center-bands only of detection of exchange (CODEX) and reference spectra of *i*P4M1P with evolution time of 2.2 ms and mixing time of 107 ms at 87 °C. (**c**) CODEX evolution time-dependence of (*S*/*S*_0_) of CH_2_ (C1) signals of *i*P4M1P with mixing time of 107 ms at 60 °C. (**d**) CODEX mixing time-dependence of (*S*/*S*_0_)* intensities for C1 (●), C2 (○), C3, (□), and C6 (×) of *i*P4M1P with evolution time of 2.2 ms at 87 °C. (Reprinted with permission from *Macromolecules* **2004**, *37*, 6460–6471 Copyright (2004) American Chemical Society).

Figure 12a depicts chemical structure and chemical shift anisotropy (CSA) of *i*P4M1P. In small chemical shift range, CSA patterns for all the functional groups overlap each other. Thereby, 2D exchange NMR based on CSA patterns cannot provide detailed dynamics information. Figure 12b represents CODEX *S* and *S*_0_ of *i*P4M1P at evolution time of 2.2 ms and mixing time of 107 ms at 87 °C, where *T*_1ρC_ relaxation filter was used to suppress the amorphous signals. CODEX data indicate that all functional groups of the crystalline chains participate in molecular dynamics at 87 °C. Figure 12c represents CODEX evolution time dependence of (*S*/*S*_0_) ratio for the backbone CH_2_ carbon (C_1_) at 60 °C. Comparisons of simulation and experimental data provide that *i*P4M1P 7_2_ helical chains perform helical jump motions at 60 °C. Mixing-time dependence of all the functional groups can be analyzed in terms of one master curve with <*τ*_c_> = 13.9 ms and *β* = 0.71, where *β* is a distribution paramter (0 < *β* ≤ 1). Both CODEX data in Figure 12c and d indicate that all the functional groups participate in helical jump dynamics in the crystalline regions. Temperature dependence of <*τ_c_*> for the helical jump motions is bent at around *T*_g_. Such unique dynamics is interpreted in terms of coupling of molecular dynamics of the amorphous and crystalline chains at the interface. Such uniqueness in molecular dynamics arises from unique densities at temperatures below 50 °C.

**Figure 13:**
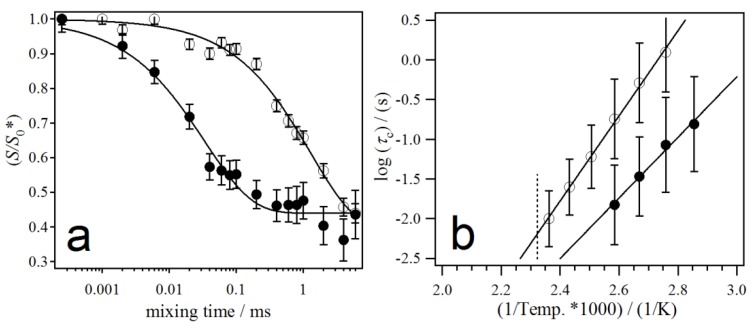
(**a**) CODEX mixing-time dependence of (*S*/*S*_0_)* signal intensities for *i*PP α_1_ (●), and the *i*PP α_2_-rich samples (○) at 95 °C. (**b**) Arrhenius plots of <*τ*_c_> of α_1_ (●), and the α_2_-rich samples (○). (Reprinted with permission from *J. Phys. Chem. B* **2010**, *114*, 92–100. Copyright (2004) American Chemical Society).

*i*PP experiences order–disorder transitions in packing structure depending on *T*_c_ as illustrated in Figure 10. It is interesting to investigate how order–disorder in packing structure affect molecular dynamics of the crystalline chain dynamics. In the literature [41], slow molecular dynamics of *i*PP α_1_- and α_2_-rich melt-grown crystals prepared at *T*_c_ = 100 and 150 °C, respectively, were investigated by CODEX NMR. <*τ*_c_> in the ordered packing structures for *i*PP α_2_ rich sample is 35 folds longer than that in disordered packing ones at the same temperature (Figure 13a). Figure 13b shows Arrhenius plots of <*τ*_c_> for the α_1_ (●) and the α_2_-rich samples (○). Using Arrhenius curves, the α_2_-rich sample show the larger activation energy of 102 ± 5 than that for the α_1_ samples (72 ± 4 kJ/mol). Tight packing of α_2_ significantly slows down mobility of *i*PP chains compared with the disordered α_1_ packing structure. It is worthwhile to note that the α_2_-rich sample shows much thicker lamellae (28 nm) than those for the α_1_ sample (11 nm). Thereby, not only packing structure but also crystal thickness contributes to slowing down the chain dynamics. The observed chain dynamics in the crystalline regions play a significant role for large deformation of semicrystalline polymers (see Section 9).

## 9. Deformation of Semicrystalline Polymers

The effects of deformation on the crystalline structure [84] and chain-level structure, for example, *R*_g_ [85,86] for semicrystalline polymers, have been successfully investigated in the past decades. However, the evolution of the locally folded chains under deformation has never been reported. 

**Figure 14:**
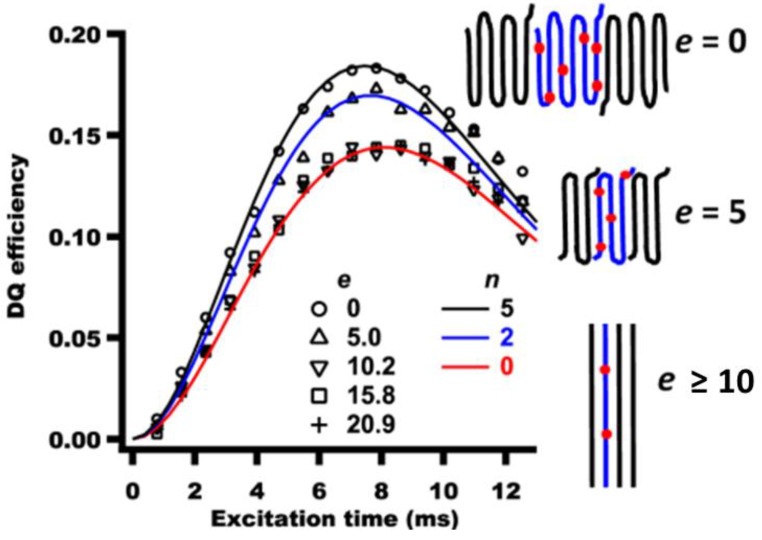
^13^C–^13^C DQ buildup curves of ^13^C 50 wt% labeled *i*PP α_1_ crystal blends with non-labeled one at a mixing ratio of 1:9 at different *e* values and calculated DQ curves with different <*n*> values. Schematic illustrations of chain-folding numbers as a function of *e*. (Reprinted with permission from *ACS Macro Lett.* **2016**, *5*, 65–68. Copyright (2016) American Chemical Society).

Kang et al. investigated chain-level structures of *i*PP α_1_ crystals under tensile drawing at 100 °C, where helical jump motions are thermally activated as shown in Figure 13b. They applied ^13^C–^13^C DQ NMR to trace <*n*> and <*F*> of *i*PP folded chains under hot drawing [60]. It is found that the folded chains are gradually unfolded with increasing engineering strain (*e*), and that the folded chain conformations are locally extended (<*n*> = 0) at *e* ≥ 10 at 100 °C (Figure 14). It is concluded that directional chain transportation occurs inside the crystallites under stretching and plays an important role for large deformability of semicrystalline polymers. Additional DQ NMR experiments on deformations at different temperatures and different stretching rates will provide complete pictures for deformation mechanisms of semicrystalline polymers at the molecular level. 

## 10. Future Subjects in ssNMR of Semicrystalline Polymers 

We reported the recent progress on understanding the chain-trajectory and molecular dynamics of various semicrystalline polymers using ssNMR. Dipolar-based NMR on ^13^C labeled sites has successfully traced chain-folding pattern and packing structure of various semicrystalline polymers. As results, it was concluded that (i) entanglement and polymer concentration significantly affect adjacent re-entry number, while available kinetics does not affect adjacent re-entry number; and (ii) polymer chains self-fold in the early stage of melt crystallization, as well as in dilute solution. The important findings falsify the well-established LH theory [3,4], and point out importance of primary nucleation in polymer crystallization [6,17]. The folded chain structures accessible by ssNMR [52,53,55,56,57,58,62] are consistent with recent views on primary nucleation in amino acids [87], metal ions [88,89], and proteins [90], among others. Combining atomic scale information by ssNMR with direct observations of morphology, as well as visualization of chains by computations, will significantly contribute to understanding in the research field of crystallization. Moreover, the novel NMR approach will be possibly applied to analysis of chain-level structure in (i) confined crystallization [91]; (ii) self-assembly at liquid/liquid interface [92,93]; (iii) other processing such as shear flow, tensile drawing, and electrospinning [94]; and (iv) glassy state.
